# A QP509L/QP383R-Deleted African Swine Fever Virus Is Highly Attenuated in Swine but Does Not Confer Protection against Parental Virus Challenge

**DOI:** 10.1128/JVI.01500-21

**Published:** 2022-01-12

**Authors:** Dan Li, Panxue Wu, Huanan Liu, Tao Feng, Wenping Yang, Yi Ru, Pan Li, Xiaolan Qi, Zhengwang Shi, Haixue Zheng

**Affiliations:** a State Key Laboratory of Veterinary Etiological Biology, Lanzhou Veterinary Research Institute, Chinese Academy of Agricultural Sciences, Lanzhou, Gansu, China; b Office international des épizooties (OIE)/National Foot and Mouth Disease Reference Laboratory, African Swine Fever Regional Laboratory of China, Lanzhou Veterinary Research Institute, Chinese Academy of Agricultural Sciences, Lanzhou, Gansu, China; Loyola University Chicago

**Keywords:** African swine fever virus, QP383R, QP509L, porcine alveolar macrophages

## Abstract

African swine fever (ASF), a devastating infectious disease in swine, severely threatens the global pig farming industry. Disease control has been hampered by the unavailability of vaccines. Here, we report that deletion of the QP509L and QP383R genes (ASFV-ΔQP509L/QP383R) from the highly virulent ASF virus (ASFV) CN/GS/2018 strain results in complete viral attenuation in swine. Animals inoculated with ASFV-ΔQP509L/QP383R at a 10^4^ 50% hemadsorbing dose (HAD_50_) remained clinically normal during the 17-day observational period. All ASFV-ΔQP509L/QP383R-infected animals had low viremia titers and developed a low-level p30-specific antibody response. However, ASFV-ΔQP509L/QP383R did not induce protection against challenge with the virulent parental ASFV CN/GS/2018 isolate. RNA-sequencing analysis revealed that innate immune-related genes (*Ifnb*, *Traf2*, *Cxcl10*, *Isg15*, *Rantes*, and *Mx1*) were significantly lower in ASFV-ΔQP509L/QP383R-infected than in ASFV-infected porcine alveolar macrophages. In addition, ASFV-ΔQP509L/QP383R-infected pigs had low levels of interferon-β (IFN-β) based on enzyme-linked immunosorbent assay (ELISA). These data suggest that deletion of ASFV QP509L/383R reduces virulence but does not induce protection against lethal ASFV challenge.

**IMPORTANCE** African swine fever (ASF) is endemic to several parts of the word, with outbreaks of the disease devastating the swine farming industry; currently, no commercially available vaccine exists. Here, we report that deletion of the previously uncharacterized QP509L and QP383R viral genes completely attenuates virulence in the ASF virus (ASFV) CN/GS/2018 isolate. However, ASFV-ΔQP509L/QP383R-infected animals were not protected from developing an ASF infection after challenge with the virulent parental virus. ASFV-ΔQP509L/QP383R induced lower levels of innate immune-related genes and IFN-β than the parental virus. Our results increase our knowledge of developing an effective and live ASF attenuated vaccine.

## INTRODUCTION

African swine fever virus (ASFV) is a complex nucleocytoplasmic large DNA virus that causes a lethal hemorrhagic disease that is currently threatening the global pig farming industry. In particular, ASFV is a large enveloped virus with a complicated architecture (1, 2), containing a double-stranded DNA genome of approximately 190 kbp, depending on the strain (3). Moreover, ASFVs are categorized into 24 different genotypes depending on the *B646L* gene, which encodes the p72 capsid protein; all genotypes have been detected in Africa (4, 5). Apart from Africa, genotype I and II ASFVs have also spread to other continents (6). Genotype I ASFV spread to Europe in the middle of the last century and was eradicated from most European countries in the 1990s (7–9). In 2007, it was found that genotype II ASFV had been introduced to Georgia, with outbreaks occurring in many European countries since then (10). Genotype II ASFV was also introduced to China and 10 other Asian countries in 2018 (11–14).

To date, no available vaccine exists for ASF, and outbreaks are currently controlled by imposing animal quarantine and the elimination of affected animals, in addition to other strict sanitary measures. In the past decades, several types of vaccines, including inactivated, DNA, subunit, and adenovirus-vector, have been evaluated for ASF, but all failed to induce protective immunity (7, 15–18). However, live attenuated ASFVs with vaccine potential owing to the specific deletion of one or more of their virulence factors have been reported to elicit protection against experimental challenge with virulent parental viruses (3, 19–23). These findings suggested that the development of attenuated ASFV recombinant viruses through the genetic manipulation of specific gene(s) could be an effective approach for vaccine development.

Following this rationale, we aimed to construct two recombinant viruses, namely, ASFV-ΔQP509L and ASFV-ΔQP383R, derived from the highly virulent ASFV CN/GS/2018 strain, by specifically deleting the QP509L or QP383R gene, respectively. We found that both ASFV-ΔQP509L and ASFV-ΔQP383R were partly attenuated *in vivo* in swine via intramuscular (i.m.) inoculation with 10^2^ 50% hemadsorbing doses (HAD_50_) (data not shown). Based on these results, here, we report the construction of a recombinant virus (ASFV-ΔQP509L/QP383R) derived from the highly virulent ASFV CN/GS/2018 isolate by specifically deleting the QP509L and QP383R genes.

## RESULTS

### Development of the QP509L and QP383R gene-deletion mutants of the ASFV CN/GS/2018 isolate.

**FIG 1:**
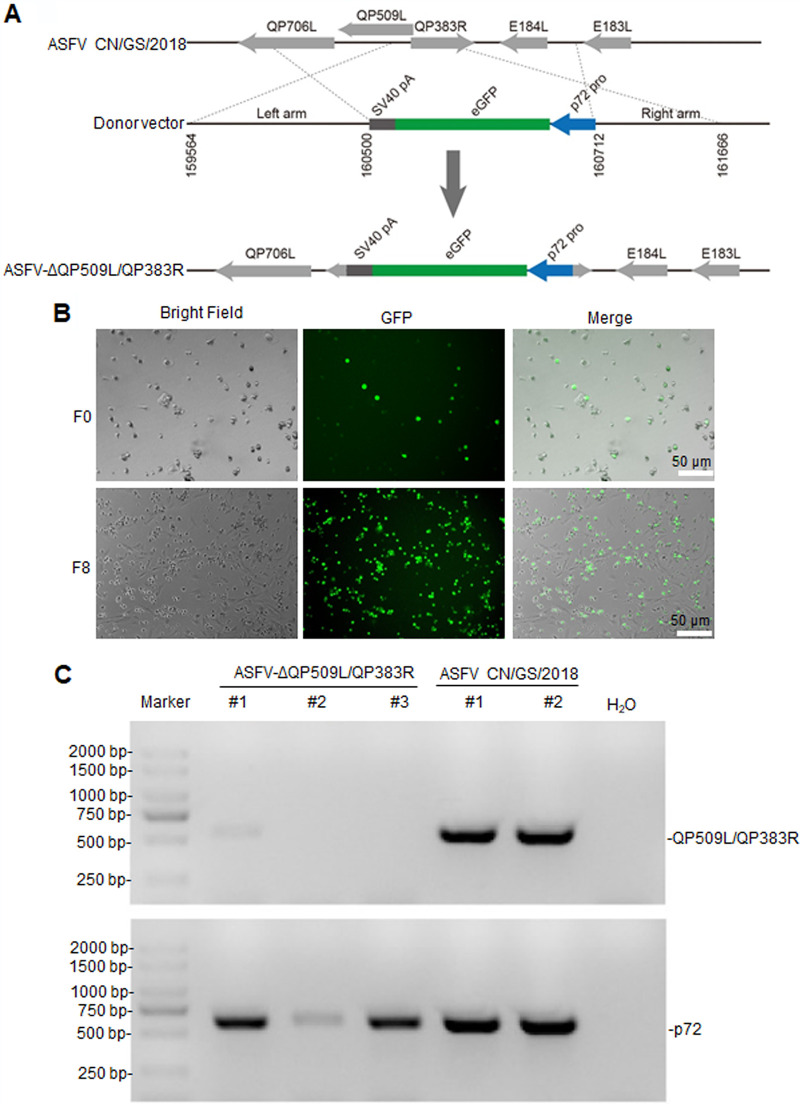
Deletion of QP509L/QP383R from the genome of the CN/GS/2018 isolate. (A) Schematic representation of the QP509L/383R gene region deleted in ASFV-ΔQP509L/383R, which was replaced with the eGFPp72 reporter gene cassette. Nucleotide positions indicating the boundaries of the deletion relative to the ASFV CN/GS/genome are indicated. (B) Virus-infected primary porcine alveolar macrophages expressing different levels of fluorescence. (C) PCR analysis of ASFV-ΔQP509L/383R stocks using specific primers targeting the QP509L/383R or p72 (B646L) genes. ASFV CN/GS/2018 DNA was used as positive control for the detection of the QP509L/383R and p72 genes. ASFV, African swine fever virus; GFP, green fluorescent protein; eGFP, enhanced GFP; SV40, simian virus 40.

Our previous research found that both ASFV-ΔQP509L and ASFV-ΔQP383R were partly attenuated in swine (data not shown). This finding prompted us to investigate whether a combined ASFV QP509L and QP383R gene-deletion mutant of the ASFV CN/GS/2018 isolate would be completely attenuated in swine. To determine the role of QP509L and QP383R in the virulence of ASFV during infection in cell culture and in swine, we designed a recombinant virus lacking the QP509L and QP383R genes (ASFV-ΔQP509L/QP383R). The ASFV-ΔQP509L/QP383R mutant was constructed from the highly pathogenic ASFV CN/GS/2018 isolate using homologous recombination procedures, as described in Materials and Methods. The gene-deleted viruses bearing one enhanced green fluorescent protein (eGFP) reporter gene were purified in primary porcine alveolar macrophages (PAMs) and confirmed by sequence analysis (Fig. 1A). The ASFV-ΔQP509L/QP383R-infected PAMs expressing green fluorescence are shown in Fig. 1B In addition, to ensure the absence of parental ASFV, viral DNA was extracted from the virus stock and analyzed by PCR using primers targeting p72 (*B646L*) and QP509L/QP383R genes. Accordingly, we found that only p72 (*B646L*) amplicons were detected in the DNA extracted from our stock; primers targeting the QP509L/QP383R genes did not generate any amplicons (Fig. 1C), indicating a lack of contamination of the ASFV-ΔQP509L/QP383R stock with parental ASFV isolate.

### Replication and growth of ASFV-ΔQP509L/QP383R in primary swine macrophages.

**FIG 2:**
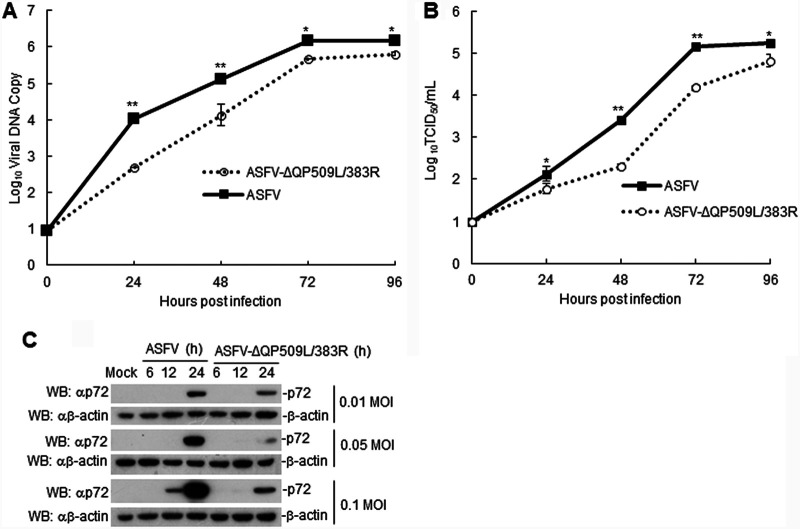
Replication of the ASFV-ΔQP509L/383R virus compared to that of the wild-type ASFV CN/GS/2018 strain. (A) Viral DNA detection in ASFV CN/GS/2018- or ASFV-ΔQP509L/383R-infected porcine alveolar macrophages (PAMs). PAMs were infected with ASFV CN/GS/2018 or ASFV-ΔQP509L/383R at a multiplicity of infection (MOI) of 0.01 for the indicated times. Samples were analyzed by qPCR. (B) Viral titers of the ASFV-ΔQP509L/383R virus, compared to those with the wild-type ASFV CN/GS/2018 strain. Viral titers recovered at different times following the infection of porcine macrophages with the ASFV CN/GS/2018 or ASFV-ΔQP509L/383R virus at an MOI of 0.01 are shown. Results from a typical experiment are shown. Viral titers in supernatants from duplicate wells were determined by an immunofluorescence assay. (C) Expression of ASFV p72 in ASFV CN/GS/2018- and ASFV-ΔQP509L/383R-infected PAMs. PAMs were infected with ASFV CN/GS/2018 or ASFV-ΔQP509L/383R (MOI: 0.01, 0.05, and 0.1) at indicated times. The samples were lysed and analyzed by Western blotting with anti-p72 or anti-actin antibodies (*n* = 3 per group; means ± SD). TCID_50_, 50% tissue culture infectious dose; WB, Western blotting.

We then evaluated the *in vitro* growth characteristics of ASFV-ΔQP509L/QP383R in cultures of primary swine macrophage cells, which are targeted by ASFV during infection in swine, and compared them to those of parental ASFV based on one-step growth curves (Fig. 2A). The results demonstrated a reduction in genomic copies of ASFV-ΔQP509L/QP383R compared to those with parental ASFV. Consistently, we found that the titers of ASFV-ΔQP509L/QP383R were approximately 10- to 80-fold lower than those of parental ASFV, depending on the time point considered (Fig. 2B). In addition, we observed that the expression of p72 in ASFV-ΔQP509L/QP383R-infected PAMs was lower than that in parental ASFV-infected PAMs with different doses of viruses (Fig. 2C).

### Assessment of ASFV-ΔQP509L/QP383R virulence in swine.

**FIG 3:**
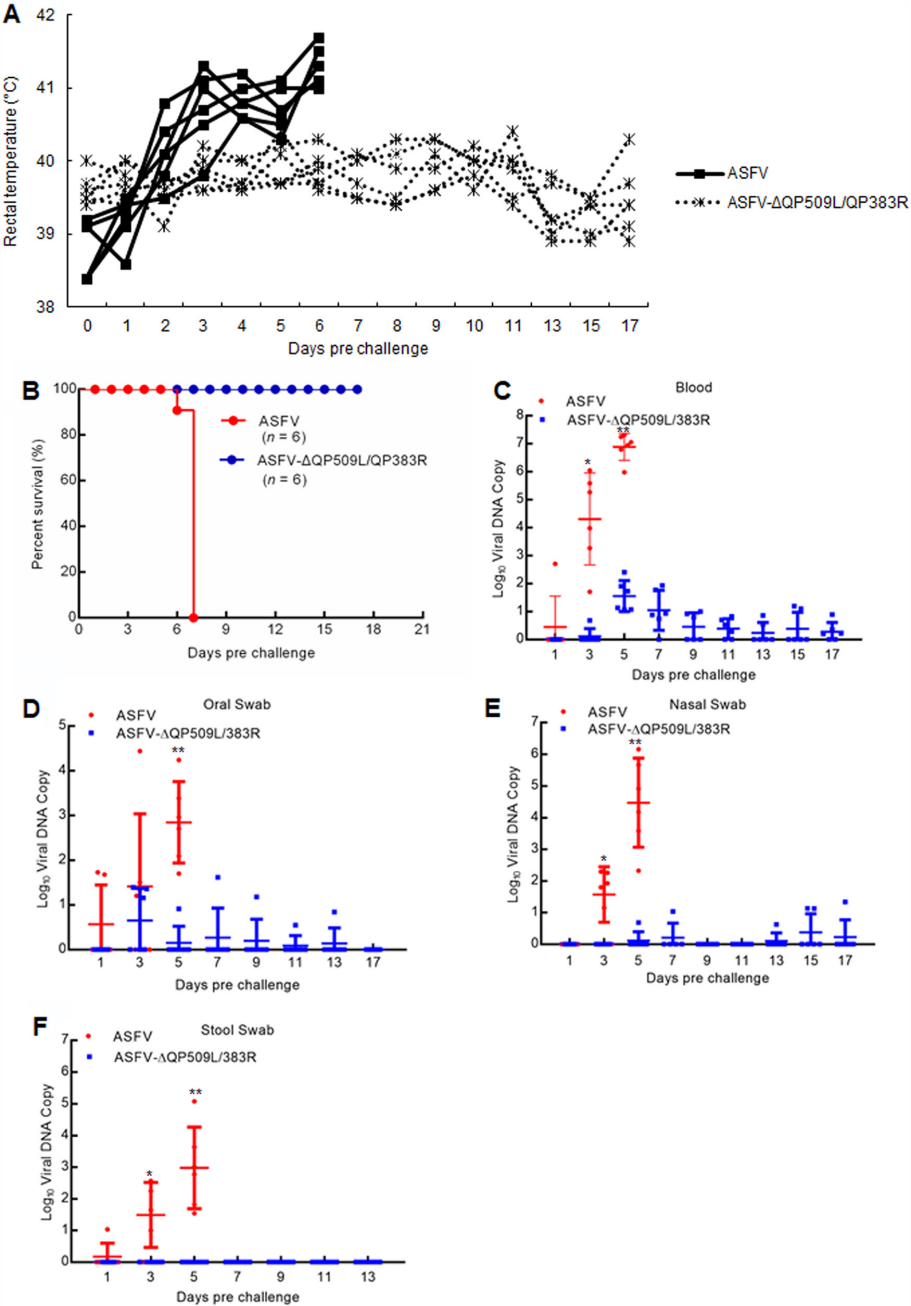
Evaluation of the virulence of QP509L/383R-deleted ASFV. (A) Kinetics of body temperature values in pigs intramuscularly (i.m.) inoculated with either 10^4^ 50% hemadsorbing dose (HAD_50_) of ASFV-ΔQP509L/383R or 10^4^ HAD_50_ of ASFV CN/GS/2018. (B) Survival rates of pigs inoculated with ASFV CN/GS/2018 and ASFV-ΔQP509L/383R with doses of 10^4^ HAD_50_. (C to F) Viral DNA detection in blood (C), oral (D), nasal (E), and stool swabs (F) of ASFV CN/GS/2018- and ASFV-ΔQP509L/383R-infected pigs. The samples were obtained from pigs infected i.m. with either 10^4^ HAD_50_ of ASFV-ΔQP509L/383R or 10^4^ HAD_50_ of ASFV CN/GS/2018 at the indicated times and analyzed by qPCR.

To investigate whether these gene-deleted viruses were attenuated in pigs, we injected (i.m.) groups of two 80- to 90-pound pigs with 10^4^ HAD_50_ of either ASFV-ΔQP509L/QP383R (*n* = 6) or ASFV CN/GS/2018 (*n* = 6) and observed the pigs for 17 days. As expected, the animals infected with 10^4^ HAD_50_ of ASFV exhibited increased body temperature (>40°C) 3 to 4 days postinfection followed by the appearance of clinical signs associated with the disease, including anorexia, depression, purple skin discoloration, staggering gait, and diarrhea. We noted that animals exhibited progressive signs of the disease over time and died within 7 days postinfection (Fig. 3A and B). Conversely, the six animals inoculated i.m. with ASFV-ΔQP509L/QP383R did not present with any ASFV infection-related signs and remained clinically normal during the entire 17-day observation period (Fig. 3A and B). Therefore, the deletion of QP509L and QP383R completely attenuated virulence in the highly virulent ASFV CN/GS/2018 isolate.

We also quantified viremia in experimentally inoculated animals at different days postinfection by quantitative real-time PCR analysis. As expected, animals inoculated with 10^4^ HAD_50_ of the virulent parental ASFV had very high viral titers in their blood until the day of their death (Fig. 3C). We found that in these groups, viremia loads reached values as high as 7.5 by the time of death. Conversely, the animals inoculated with 10^4^ HAD_50_ of mutant ASFV-ΔQP509L/QP383R had relatively low viral titers in their blood compared with those of parental ASFV-inoculated animals. Moreover, the animals inoculated with 10^4^ HAD_50_ of mutant ASFV-ΔQP509L/QP383R had a heterogeneous pattern of viral titers in their blood. Only one animal carried viremia DNA copies that reached 2.0 at approximately day 6 (Fig. 3C). In addition, we also detected viral DNA copies in oral (Fig. 3D), nasal (Fig. 3E), and stool swabs (Fig. 3F). The results showed that animals inoculated with 10^4^ HAD_50_ of mutant ASFV-ΔQP509L/QP383R had relatively few viral DNA copies in oral, nasal, and stool swabs compared with those in parental ASFV-inoculated animals. Altogether, the animals infected with ASFV-ΔQP509L/QP383R tended to present with lower viral titers in their blood and oral, nasal, and stool swabs relative to those in animals inoculated with parental ASFV.

### ASFV-ΔQP509L/QP383R virus does not provide protection against challenge with the virulent parental virus.

We next aimed to assess the effect of ASFV-ΔQP509L/QP383R inoculation on the induction of host protection against ASFV infections. To this end, ASFV-ΔQP509L/QP383R-exposed animals were challenged with the virulent parental ASFV CN/GS/2018 isolate. The group of pigs inoculated with 10^4^ HAD_50_ of ASFV-ΔQP509L/QP383R was then challenged i.m. at 17 days postinfection with 10^2^ HAD_50_ of the virulent parental ASFV. The animals were monitored daily for clinical signs and changes in body temperature.

**FIG 4:**
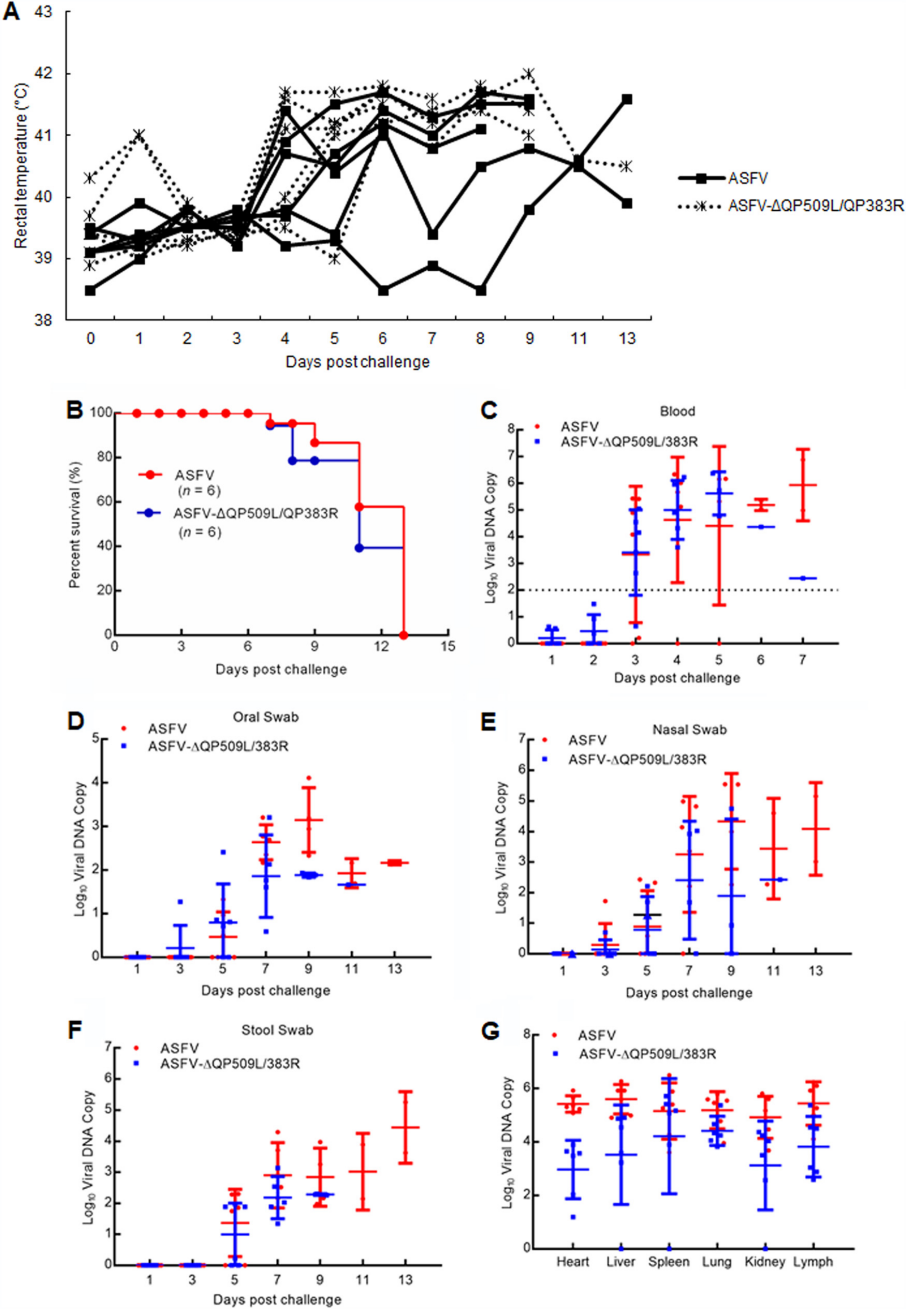
Protective efficacy induced by the QP509L/383R-deleted ASFV in pigs. (A) Temperatures at different days postchallenge with ASFV CN/GS/2018 at 10^2^ 50% hemadsorbing dose (HAD_50_). (B) Survival rates of pigs. Pigs inoculated with 10^4^ HAD_50_ of QP509L/383R-deleted ASFV were challenged intramuscularly (i.m.) with lethal ASFV CN/GS/2018 at a dose of 10^2^ HAD_50_. (C to G) Viral DNA detection in blood (C), oral (D), nasal (E), stool swabs (F), and different tissues (G) of pigs. The experiments were performed similarly to the experiment in panel B. Viral DNA was analyzed by qPCR.

Six naive animals that were challenged with parental ASFV using the same route and dose served as the control group. These animals displayed ASF-related signs at 4 days postchallenge (dpc), with clinical signs evolving into more-severe manifestations in the following days, and all of the animals died within 13 dpc (Fig. 4A and B). Kinetics of the appearance of clinical signs, including the increase in body temperature, was similar to those of control animals challenged with ASFV. Moreover, we did not detect any significant differences in the duration of disease or the time at which animals were euthanized between the animals previously infected with ASFV-ΔQP509L/QP383R and those challenged with the virulent parental ASFV (Fig. 4A). Conclusively, all of the animals receiving 10^4^ HAD_50_ of ASFV-ΔQP509L/QP383R were shown to succumb to challenge with the virulent parental virus (Fig. 4B).

The presence of viral DNA copies in the blood upon challenge was demonstrated to be an indicator of the protective potency of the immunity elicited by ASFV-ΔQP509L/QP383R. We also detected postchallenge viremia in these animals. Challenged control animals infected with the virulent parental ASFV presented with viral DNA copies (approximately 4.0) after 5 days, increasing (to approximately 6.0) by 7 to 9 days. After challenge with the virulent parental ASFV, levels in ASFV-ΔQP509L/QP383R-infected animals did not differ from those in challenged control animals (Fig. 4C). Consistently, the viral DNA copies in the oral, nasal, and stool swabs of ASFV-ΔQP509L/QP383R-infected animals after the challenge with the virulent ASFV were also not different from those of challenged control animals (Fig. 4D to F). In addition, the viral DNA copies in the heart, liver, spleen, kidney, and lymph of ASFV-ΔQP509L/QP383R-infected animals were not significantly different from those in parental ASFV-infected animals (Fig. 4G). Collectively, these data suggested that ASFV-ΔQP509L/QP383R does not protect against ASFV CN/GS/2018 homologous lethal challenge.

### RNA-Seq-based transcriptome analysis of ASFV-ΔQP509L/QP383R- and ASFV-infected PAMs.

**FIG 5:**
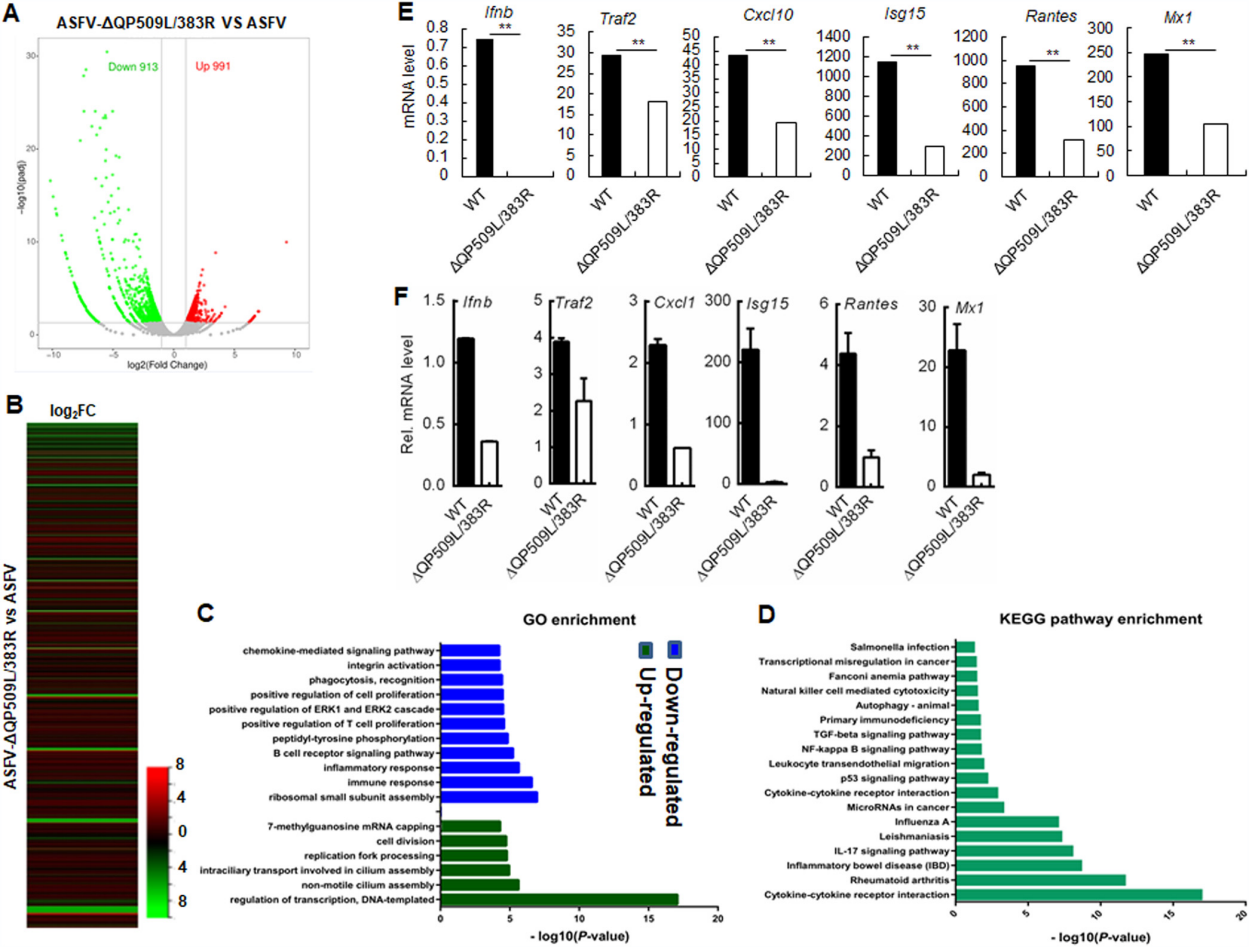
RNA-Seq analysis to identify QP509L/383R-deleted ASFV-regulated genes in PAMs at 24 h. (A) Volcano map of differentially expressed genes (DEGs) of DRG in ASFV- versus ASFV-ΔQP509L/383R-infected PAMs at 24 h. Red dots indicate significantly upregulated DEGs; green dots indicate significantly downregulated DEGs; gray dots indicate no significance. (B) Heat map of DEGs of DRG between ASFV- and ASFV-ΔQP509L/383R-infected PAMs at 24 h. (C) GO terms. (D) KEGG pathway enrichment. (E) Expression level of *Ifnb*, *Traf2*, *Cxcl10*, *Isg15*, *Rantes*, and *Mx1* genes in ASFV-ΔQP509L/383R- and ASFV-infected PAMs by RNA-Seq analysis at 24 h. (F) Expression level of *Ifnb*, *Traf2*, *Cxcl10*, *Isg15*, *Rantes*, and *Mx1* genes in ASFV-ΔQP509L/383R- and ASFV-infected PAMs by qPCR analysis. PAMs were infected with ASFV or ASFV-ΔQP509L/383R (multiplicity of infection [MOI]: 0.1) for 24 h. The samples were analyzed by qPCR (*n* = 3 per group; means ± SD). ERK, extracellular signal-regulated kinase; IL-17, interleukin-17; TGF-beta, transforming growth factor-beta; WT, wild type (ASFV).

Volcano maps showed that there were 913 downregulated and 991 upregulated differentially expressed genes (DEGs) between the ASFV- and ASFV-ΔQP509L/QP383R-infected PAMs at 24 h (Fig. 5A). The heat map of DEGs between the ASFV- and ASFV-ΔQP509L/QP383R-infected PAMs at 24 h is presented in Fig. 5B The gene ontology (GO) analysis showed that upregulated and downregulated DEGs in ASFV-ΔQP509L/QP383R-infected PAMs were enriched in GO biological process terms mainly related to regulation of transcription and nonmotile cilium assembly, ribosomal small subunit assembly, and immune mechanisms, respectively (Fig. 5C). The DEGs between the ASFV- and ASFV-ΔQP509L/QP383R-infected PAMs were enriched in 17 pathways by KEGG analysis (Fig. 5D). Previous studies have shown that IFN-β, TRAF2, CXCL10, RANTES, ISG15, and MX1 have important roles in the antiviral response (24–29). RNA-sequencing (RNA-Seq) verified that the expression levels of the *Ifnb*, *Traf2*, *Cxcl10*, *Isg15*, *Rantes*, and *Mx1* genes were downregulated in ASFV-ΔQP509L/QP383R-infected PAMs compared with those in ASFV-infected PAMs (Fig. 5E). In addition, quantitative PCR (qPCR) also confirmed that these genes were downregulated in ASFV-ΔQP509L/QP383R-infected PAMs compared with levels in ASFV-infected PAMs (Fig. 5F).

### Host antibody response in animals infected with ASFV-ΔQP509L/QP383R.

**FIG 6:**
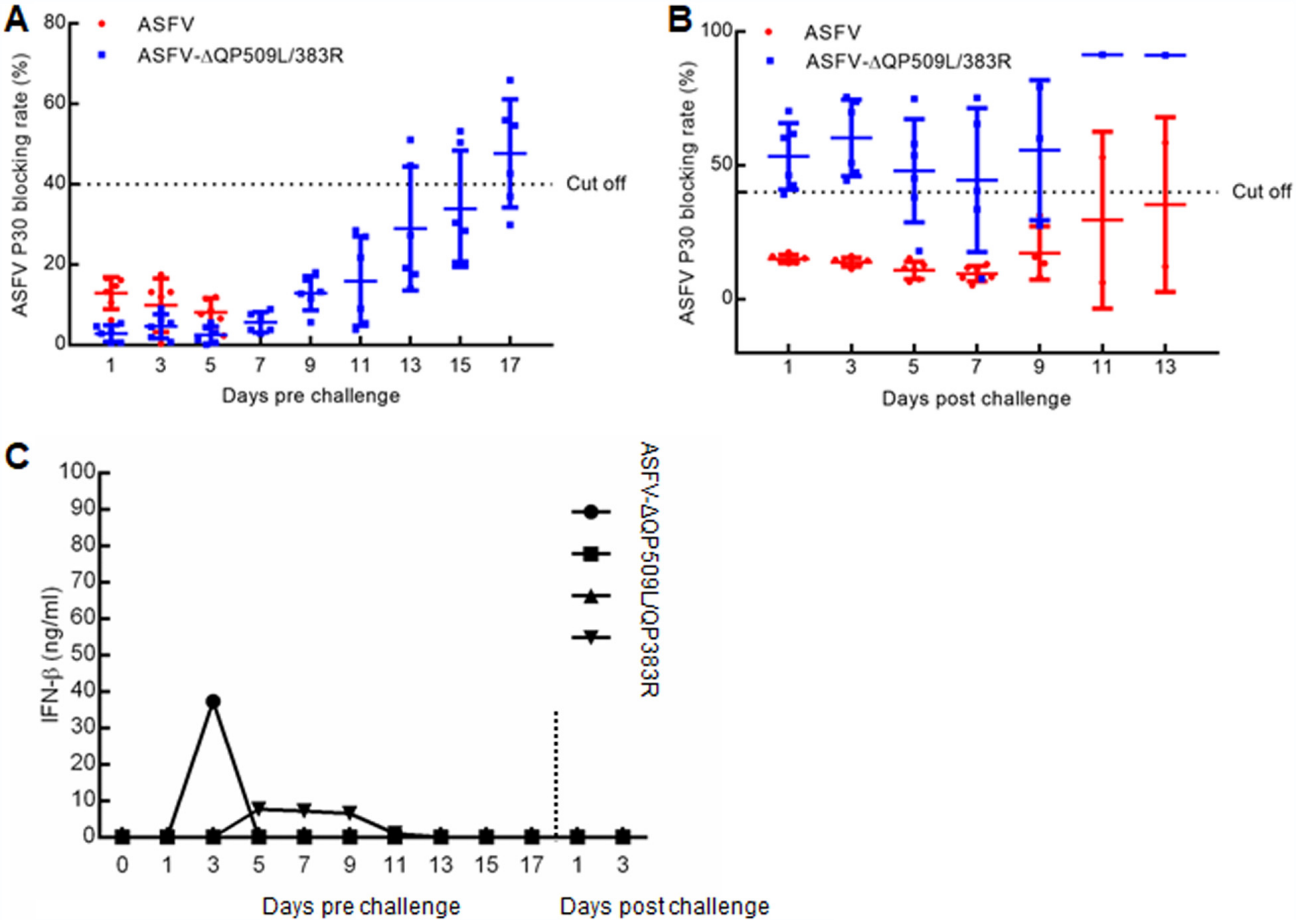
Immune responses detected following immunization with ASFV-ΔQP509L/383R and challenge with ASFV CN/GS/2018. (A, B) Levels of ASFV p30-specific antibodies measured using a blocking ELISA on the indicated days postimmunization (A) and postchallenge (B) (*x* axis). The values for individual pigs are shown. (C) Levels of interferon-β (IFN-β) detected in the serum by ELISA at different days postimmunization and postchallenge (*x* axis). The results for individual pigs are shown.

Whether the host immune mechanisms mediate protection against virulent strains of ASFV in animals infected with attenuated strains of the virus is not well known (30–32). Previous studies have indicated that the only parameter consistently associated with protection against challenge is the level of circulating antibodies (3). To detect the antibodies in ASFV-ΔQP509L/QP383R-infected animals, we attempted to correlate the presence of anti-p30 circulating antibodies with protection. Our results showed that the ASFV p30-blocking rate was <40% in most of ASFV-ΔQP509L/QP383R-infected animals until 16 days, and the ASFV p30-blocking rate was >40% in several ASFV-ΔQP509L/QP383R-infected animals at 12 to 17 days, whereas levels of the p30 antibody in ASFV-infected animals did not change until death (Fig. 6A). We noted that with the exception of a few animals at days 5 to 10, most animals challenged with the highly the virulent parental ASFV isolate had high levels of the p30 antibody, whereas control animals had low levels of the p30 antibody, except for one animal on days 11 to 13 (Fig. 6B). To explore whether ASFV-ΔQP509L/QP383R induced a host immune response, we detected the expression of IFN-β in the sera of pigs. We observed that two of the four ASFV-ΔQP509L/QP383R-infected pigs exhibited low levels of IFN-β expression at days 3 and 5 to 9, respectively (Fig. 6C). Altogether, these results suggested that ASFV-ΔQP509L/QP383R induced a low-level immune response *in vivo*.

## DISCUSSION

The use of live attenuated ASFV strains is currently the most plausible approach to effectively protect pigs against challenge with homologous virulent isolates. In general, live attenuated ASFV strains can be obtained via sequential passage in cell culture or genetic manipulation (33–35). Several attenuated strains, generated by deleting single genes or a group of genes, have been evaluated as potential vaccines (3, 19–23). For example, deletion of the DP148R gene was reported to attenuate the virulent Benin 97/1 isolate but not the HLJ/18 virus in pigs (19, 23). Similarly, deletion of the CD2v gene was shown to highly attenuate the virulent BA71 strain but not the HLJ/18 virus or the Georgia/2010 strain *in vivo* (23, 36, 37). Interestingly, deletion of the 9GL and UK genes in the ASFV Georgia/2007 isolate was demonstrated to be safe and protected pigs against homologous virus challenge. However, deletion of the same genes in the HLJ/18 virus did not provide any protection against homologous virus challenge, despite leading to the attenuation of the virus in pigs (20, 23). These findings suggest that the same genes might have different functions in different ASFV strains.

The ASFV QP509L gene, which is known to play a key role in viral replication and transcription, is an RNA helicase highly conserved among virulent and nonvirulent isolates (38). We found that deletion of the QP509L gene partly attenuated the virulent CN/GS/2018 virus (data not shown). Likewise, deletion of the ASFV QP383R gene, which is known to inhibit the inflammatory response (39), only partly attenuated the virulent CN/GS/2018 virus (data not shown). In this study, we demonstrated that an ASFV CN/GS/2018 isolate (a genotype II strain) lacking the QP509L and QP383R genes (double-deletion mutant) was completely attenuated *in vivo* in swine inoculated i.m. with 10^4^ HAD_50_, as pigs remained healthy, without signs of the disease. However, ASFV-ΔQP509L/QP383R did not induce protection against challenge with the virulent parental ASFV strain. In addition, deletion of the QP509L and QP383R genes affected the ability of the virus to replicate in primary swine macrophage cultures compared to that with the corresponding parental virus.

Previous studies indicated that the deletion of some ASFV genes leads to the attenuation of the virus in pigs but does not provide any protection against homologous virus challenge (23, 40); however, there has been no study on the mechanism underlying this effect. Here, we found that deletion of the QP509L/QP383R genes in ASFV CN/GS/2018 induced low levels of IgG and IgM in the host (data not shown). A previous study showed that deletion of I177L could induce protection against the ASFV Georgia isolate and lead to the generation of a strong IgG and IgM response in pigs (3). In addition, we found that deletion of QP509L/QP383R genes in ASFV CN/GS/2018 induced a low-level type I interferon response using RNA-Seq and enzyme-linked immunosorbent assay (ELISA).

We also demonstrated that the replication of the mutant ASFV-ΔQP509L/QP383R was significantly decreased compared with that of the parental ASFV CN/GS/2018 isolate. We assume that a low- or no-replication phenotype of live attenuated ASFV strains used as effective ASF vaccines at late stages of infection would play an important role in vaccine safety. However, we found that ASFV-ΔQP509L/QP383R induced an ASFV p30 antibody response at very late stages of infection. Consistently, ASFV-ΔQP509L/QP383R was also shown to induce lower levels of type I interferon than those with parental ASFV. Therefore, we speculated that deletion of the QP509L and QP383R genes highly attenuated the virulent CN/GS/2018 strain (a genotype II virus) *in vivo* owing to the low replication of the mutant ASFV-ΔQP509L/QP383R. ASFV-ΔQP509L/QP383R did not provide protection against challenge with virulent ASFV, possibly due to the low levels of type I interferon gene expression, which constitutes the ASFV-specific antibody response at very late stages of infection, but the exact mechanism needs to be further studied. In summary, we used the Chinese ASFV CN/GS/2018 isolate as a backbone to generate a virus bearing a double gene deletion and found that ASFV-ΔQP509L/QP383R was fully attenuated in pigs but could not induce complete protection against lethal ASFV challenge.

## MATERIALS AND METHODS

### Ethics statements.

This study was carried out in strict accordance with the recommendations provided in the Guide for the Care and Use of Laboratory Animals of the Ministry of Science and Technology of the People’s Republic of China. All protocols were approved by the Committee on the Ethics of Animal Experiments of the Lanzhou Veterinary Research Institute (LVRI) of the Chinese Academy of Agricultural Sciences (CAAS) and the Animal Ethics Committee of Lanzhou Province, China.

### Biosafety statement and facility.

All experiments using live ASF viruses were conducted within the enhanced biosafety level 3 (P3) facilities in the LVRI of the CAAS and approved by the Ministry of Agriculture and Rural Affairs and the China National Accreditation Service for Conformity Assessment.

### Cell culture and viruses.

PAMs were prepared by bronchoalveolar lavage as previously described (41) and grown in Dulbecco’s modified Eagle’s medium (DMEM) supplemented with 2 mM l-glutamine, 100 U/ml gentamicin, nonessential amino acids, and 10% porcine serum. The cells were grown at 37°C in a 7% CO_2_ atmosphere saturated with water vapor. The CN/GS/2018 ASFV isolate was provided by the African Swine Fever Regional Laboratory of the Lanzhou Veterinary Research Institute and propagated in PAMs. Briefly, subconfluent PAM cells were cultivated in p150 plates and infected with ASFV at a multiplicity of infection (MOI) of 0.2 PFU/cell in DMEM supplemented with 10% porcine serum. At 96 h postinfection (hpi), the cells were recovered and centrifuged at 3,000 rpm for 15 min. The cell pellet was discarded. The supernatant containing the viruses was clarified at 14,000 rpm for 6 h at 4°C, resuspended in medium, and stored at −80°C. Infection was performed after ASFV viral adsorption at 37°C for 90 min, when the inoculum was removed, and fresh medium was added. The cells were then incubated at 37°C until the indicated hpi. The ASFV CN/GS/2018 isolate lacking the QP509L and QP383R genes was named ASFV-ΔQP509L/QP383R.

### qPCR.

ASFV genomic DNA was extracted from cell supernatants, tissue homogenate, or EDTA-treated whole peripheral blood using GenElute mammalian genomic DNA miniprep kits (Sigma-Aldrich, USA). qPCR was carried out using a QuantStudio 5 system (Applied Biosystems, USA) according to the Office international des épizooties (OIE)-recommended procedure.

### RNA-Seq.

PAMs were infected with ASFV CN/GS/2018 or ASFV-ΔQP509L/383R for 24 h. Total RNA was extracted as previously described (42). The quality of total RNA with an RNA integrity number of 7 or higher was assessed using an Agilent 2100 Bioanalyzer. Illumina TruSeq RNA libraries were constructed from two total RNA samples (each sample was a mixture of three biological repeats). The libraries were sequenced using 100-bp paired-end reads on an Illumina HiSeq 2000. Sequenced reads were checked for base qualities, trimmed wherein 20% of bases were below a quality score of 20, and filtered to exclude sequences that were shorter than 20 bp using Fastx (Version 0.0.13). Sequences were aligned to the *Xenopus tropicalis* genome JGI 4.2/xenTro3 using gsnap (43) with the following parameters: -B 4-E 100 -N 1. The aligned reads were counted using HTSeq (version 0.5.4p2) with the following parameters: -m intersection-strict -s no -a 20, and further differential gene expression analysis was carried out using DESeq2 (version 1.0.19) (44) with default settings.

### Generation of QP509L/QP383R-deficient virus.

The gene-deleted ASFV QP509L/383R mutant was generated via homologous recombination between the genome of the parental ASFV CN/GS/2018 isolate and recombination transfer vectors through transfection and infection procedures. The pUC19 plasmid lacking multiple cloning sites was used as a backbone, and a recombination cassette was inserted at the SalI and NdeI restriction sites after the T7 promoter. The recombination cassette contained a left recombination arm located at positions 159564 to 160500 in the genome of ASFV CN/GS/2018, followed by eGFP and a simian virus 40 (SV40) termination sequence, followed by a right recombination arm located at positions 160712 to 161666 in the genome of ASFV CN/GS/2018. Recombinant transfer vectors were constructed using fusion PCR and the Gibson assembly technique (Invitrogen Life Sciences, USA). PAMs were transfected with transfer vectors using the TransITLT1 transfection reagent (Mirus Bio, Madison, WI) and then infected with ASFV CN/GS/2018 at 24 h posttransfection. The DNA fragment of QP509L/QP383R was deleted from the genome of the ASFV CN/GS/2018 isolate via homologous recombination, and the resulting virus was purified by successively picking fluorescent plaques combined with limited dilution on monolayers of PAMs. The virus obtained from the last round of purification was amplified in PAMs to generate a viral stock. To ensure the absence of parental CN/GS/2018 and the presence of desired deletions in the recombinant genome, DNA was extracted from the viral stock and confirmed by PCR analysis (using the primers ccgcccgctgatagtttttc [forward] and cgaggagtgatggtatctaaatgc [reverse]) and sequencing.

### Viral titration.

The wild-type ASFV CN/GS/2018 and QP509L/383R-deficient viruses were quantified using the hemadsorption (HAD) assay as previously described (45) with minor modifications. Briefly, PAM cells were seeded in 96-well plates. Viral samples were then added to the plates and titrated in triplicate using 10-fold serial dilutions. HAD was determined on day 7 postinoculation, and HAD_50_ values were calculated using the method described by Reed and Muench (46).

Samples from the ASFV-ΔQP509L/383R-infected cell supernatants were quantified by testing their 50% tissue culture infectious dose (TCID_50_). PAMs were seeded into 96-well plates, and 3 days later, 10-fold serially diluted samples were added into each well in triplicate. After culturing for 7 days, the expression of the fluorescent protein was assessed using fluorescence microscopy. The TCID_50_ values were calculated using the method described by Reed and Muench (46).

### Animal experiments.

The virulence and pathogenesis of ASFV-ΔQP509L/QP383R, relative to those of the parental ASFV CN/GS/2018 virus, were assessed using 80- to 90-pound commercial breed swine. Twelve pigs were inoculated (i.m.) with either 10^4^ HAD_50_ of ASFV-ΔQP509L/QP383R or ASFV CN/GS/2018. The pigs were monitored daily for 17 days for temperature, clinical signs, and mortality. Blood, oral swabs, stool swabs, and nasal swabs collected at 1, 3, 5, 7, 9, 11, 13, 15, and 17 dpc were used for the detection of viral DNA and the expression of cytokines and antibodies against p30. In protection experiments, animals inoculated with ASFV-ΔQP509L/QP383R were challenged i.m. 17 days later with 10^2^ HAD_50_ of virulent parental ASFV strain. Viral loads were determined by qPCR as recommended by the OIE-recommended procedure. The target for amplification of the ASFV genome was the conserved p72 gene segment, using the following primers: 5′-ctgctcatggtatcaatcttatcga-3′ and 5′-gataccacaagatc(ag)gccgt-3′. A TaqMan probe (5′-[6-carboxyfluorescein (FAM)]-ccacgggaggaataccaacccagtg-3′-[6-carboxytetramethylrhodamine (TAMRA)] from Applied Biosystems) was designed from the alignment of 54 available ASFV sequences for the 3′-end of p72 (47). The expression levels of serum IFN-β was determined by ELISA using an ELISA kit (Solarbio Life Sciences, SEKP-0046, SEKP-0012, SEKP-0014) according to the manufacturer’s instructions. Detection of the ASFV p30 antibody was performed with an in-house blocking ELISA.

### Blocking ELISA.

ELISA microtiter plates were coated using an optimal concentration (8 μg/ml) of p30 protein (100 μl/well) in 0.05 M carbonate buffer solution (pH 9.6) and incubated overnight at 4°C. Antigen-coated plates were washed three times with PBST (PBS containing 0.5% [vol/vol] Tween 20), and nonspecific binding sites were blocked with 200 μl of blocking buffer (2.5% [wt/vol] nonfat dry milk in PBST) overnight at 4°C. After three washes with PBST, 100 μl of test samples, positive serum samples, and negative serum samples, diluted (1:2) in blocking buffer, were separately added to each well in duplicate. Next, the plates were incubated for 30 min at 37°C followed by three washes, the addition of 100 μl/well of p30 MAb-HRP (1:25,000), and incubation at 37°C for an additional 30 min. Following a final triplicate wash, 100 μl/well of 3,3′,5,5′-tetramethylbenzidine (TMB) substrate prepared by mixing A and B solutions (A: 205 mM potassium citrate, pH 4.0; B: 41 mM TMB) at a 39:1 ratio (A:B, vol/vol) was added to each well, and the plates were incubated in the dark for 15 min at 37°C. As a final step, 2 M H_2_SO_4_ (50 μl/well) was used to stop the colorimetric reaction, and the optical density at 450 nm (OD_450_) values were read using an automated ELISA plate reader.

### Statistical analysis.

Each experiment shown is representative of three replicate experiments performed. All data are expressed as the means ± standard deviation (SD). An unpaired two-tailed Student’s *t* test was used for statistical analysis.
